# A Combined ELONA-(RT)qPCR Approach for Characterizing DNA and RNA Aptamers Selected against PCBP-2

**DOI:** 10.3390/molecules24071213

**Published:** 2019-03-28

**Authors:** Miguel Moreno, María Fernández-Algar, Javier Fernández-Chamorro, Jorge Ramajo, Encarnación Martínez-Salas, Carlos Briones

**Affiliations:** 1 Laboratory of Molecular Evolution, Centro de Astrobiología (CSIC-INTA), Torrejón de Ardoz, 28850 Madrid, Spain; morenomm@cab.inta-csic.es (M.M.); fernandezam@cab.inta-csic.es (M.F.-A.); 2 Centro de Biología Molecular “Severo Ochoa” (CSIC-UAM), 28049 Madrid, Spain; jfernandez@cbm.uam.es (J.F.-C.); jramajo@cbm.uam.es (J.R.); emartinez@cbm.csic.es (E.M.-S.); 3 Centro de Investigación Biomédica en Red de Enfermedades Hepáticas y Digestivas (CIBERehd), 28029 Madrid, Spain

**Keywords:** aptamer, SELEX, in vitro selection, ELONA, ELASA, qPCR, RTqPCR, PCBP-2

## Abstract

Improvements in Systematic Evolution of Ligands by EXponential enrichment (SELEX) technology and DNA sequencing methods have led to the identification of a large number of active nucleic acid molecules after any aptamer selection experiment. As a result, the search for the fittest aptamers has become a laborious and time-consuming task. Herein, we present an optimized approach for the label-free characterization of DNA and RNA aptamers in parallel. The developed method consists in an Enzyme-Linked OligoNucleotide Assay (ELONA) coupled to either real-time quantitative PCR (qPCR, for DNA aptamers) or reverse transcription qPCR (RTqPCR, for RNA aptamers), which allows the detection of aptamer-target interactions in the high femtomolar range. We have applied this methodology to the affinity analysis of DNA and RNA aptamers selected against the poly(C)-binding protein 2 (PCBP-2). In addition, we have used ELONA-(RT)qPCR to quantify the dissociation constant (*Kd*) and maximum binding capacity (*Bmax*) of 16 high affinity DNA and RNA aptamers. The *Kd* values of the high affinity DNA aptamers were compared to those derived from colorimetric ELONA performed in parallel. Additionally, Electrophoretic Mobility Shift Assays (EMSA) were used to confirm the binding of representative PCBP-2-specific RNA aptamers in solution. We propose this ELONA-(RT)qPCR approach as a general strategy for aptamer characterization, with a broad applicability in biotechnology and biomedicine.

## 1. Introduction

Aptamers are single-stranded (ss) DNA or RNA molecules that can bind with high affinity and specificity to a desired target molecule [1]. They are isolated from a large library of synthetic random oligonucleotides using an amplification-selection in vitro process termed Systematic Evolution of Ligands by EXponential enrichment (SELEX) [2]. Aptamers adopt a specific three-dimensional solution structure that depends on their sequence and the physicochemical features of the folding buffer (including the temperature, ionic strength, and concentration of divalent cations). The selected DNA and RNA aptamers are able to recognize and, eventually, alter the activity of their target molecules by establishing non-covalent aptamer–target molecular interactions. The current SELEX technology allows the generation of aptamers with high affinity and specificity for a broad range of targets, including low molecular weight compounds, peptides, proteins, structured nucleic acids, macromolecular complexes, and even whole cells [3]. As a result, aptamers have being used in a growing number of biotechnological and biomedical applications over the last decade [4].

Next generation sequencing (NGS) of DNA is currently replacing the traditional methods based on the analysis of clonal sequences, which provides a significant information increase on the selected pools of aptamers [5,6]. Consequently, a vast number of selected, putative aptamer sequences are obtained at the end of any SELEX process and, therefore, the screening of those showing the highest affinity for the target has become more complex and time-consuming. Sequence alignments can be performed using a wide range of bioinformatics tools, such as Clustal Omega, Multiple Alignment using Fast Fourier Transform (MAFFT), and T-Coffee [7,8], while others specifically devoted to the aptamer field, including FastAptamer [6], AptaCluster [9], or AptaGUI [10], have recently been developed. Based on their output, aptamer candidates can be grouped in various families or categories, further subject to secondary structure prediction using web servers, such as RNAfold [11] or Mfold [12]. 

A considerable number of individual aptamer candidates (from dozens to even hundreds) must be functionally screened for their binding affinity and specificity against the cognate target. The quantitative measurement of the aptamer–target binding reaction is often calculated by means of the dissociation constant of the aptamer (*Kd*) and its maximum binding capacity (*Bmax*). Over the last two decades, several methods have been used to characterize the binding affinity of aptamers, all of them showing merits and limitations. Among them, colorimetric or fluorescence-based techniques, Electrophoretic Mobility Shift Assays (EMSA), or those depending on additional biophysical principles (surface plasmon resonance, thermal shift assays, circular dichroism, or UV-vis melting profiles, to name a few) are the preferred methodologies [13,14,15]. However, most of them are laborious and unworkable for analysing large numbers of individual aptamers. Additionally, the above-mentioned techniques have been optimized to characterize either DNA or RNA aptamers, but not both types of molecules in parallel. 

Therefore, simpler, faster, and cost effective methods are required for the functional characterization of aptamers. Drolet and coworkers [16] developed a modification of the traditional Enzyme-Linked Immuno Sorbent Assay (ELISA) [17] by using aptamers instead of antibodies, which they termed Enzyme-Linked OligoNucleotide Assay (ELONA). The original method, also known as Aptamer-Linked Immobilized Sorbent Assay (ALISA) [18] or Enzyme-Linked Aptamer Sorbent Assay (ELASA) [19], employed a fluorescent or colorimetric readout and allowed the simultaneous analysis of numerous aptamers. However, the use of fluorescence-labelled DNA or RNA aptamers is an expensive option for the characterization of large populations. Apart from that, colorimetric readout is easily adaptable to DNA aptamers (through biotin or digoxigenin labelling), but it is less optimized for RNA aptamers (because of the inherent technical difficulties associated to RNA labelling) [20,21,22]. Furthermore, colorimetric reactions generally show a limited sensitivity and require additional, time-consuming recognition reactions involving either streptavidin or an anti-digoxigenin antibody labelled with horseradish peroxidase (HRP) or alkaline phosphatase (AP) [23]. To overcome such limitations, here, we combine the mid-range sample throughput potential of ELONA with the sensitivity of SYBR green-based, real-time quantitative PCR(qPCR). The use of aptamers as (RT)qPCR-amplifiable reporter molecules excludes the need for aptamer or target labelling, allowing a straightforward assay with enhanced sensitivity. Hence, ELONA-qPCR and, for the first time, ELONA-RTqPCR were used to functionally analyse DNA and RNA aptamers in parallel. This provides a significant advance to the post-SELEX aptamer characterization. 

**Figure 1:**
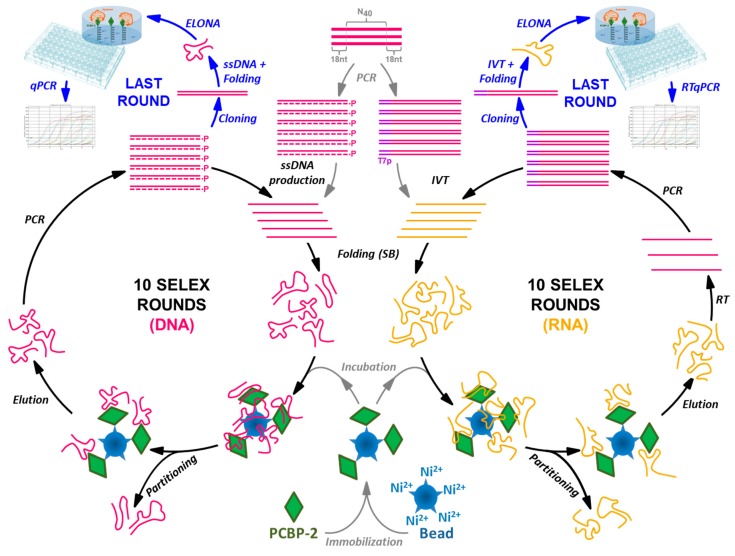
Schematic representation of the SELEX processes performed to select ssDNA and RNA aptamers in parallel using PCBP-2 as the target molecule, coupled to the ELONA-(RT)qPCR methodology developed for the functional characterization of the individual aptamers present at the last SELEX round.

In this work, the combined features of ELONA-(RT)qPCR were applied to the characterization of nucleic acid populations obtained after 10 rounds of DNA and RNA aptamer selection performed in parallel against poly(C)-binding protein 2 (PCBP-2) (Figure 1). PCBP-2 was chosen as the target molecule for this study due to the biological relevance of this pleiotropic protein, for which aptamers have not been selected before. Our experimental setup allowed the analysis of more than 50 DNA and RNA individual aptamers, all of them obtained after molecular cloning and sequencing of their respective last selection round. Thereafter, the aptamers showing the highest affinity to PCBP-2 were characterized by means of their *Kd* and *Bmax* calculation, and some of them were revealed as high affinity molecular probes that could be used for the development of highly sensitive aptamer-based biosensors or ‘aptasensors’. As a validation technique, colorimetric ELONA was used in parallel to quantify the *Kd* values of the high affinity DNA aptamers. Additionally, EMSA was employed to confirm that representative examples of the high affinity RNA aptamers were able to form complexes with the target protein in solution. 

## 2. Results

### 2.1. DNA and RNA Aptamer Selection

Target PCBP-2 was expressed as a fusion protein with a polyhistidine (6xHis) tag at its N-terminal end, thus allowing its immobilization onto Cu^2+^-coated polystyrene plates (Supplementary Figure S1), as well as on beaded agarose derivatized with the nitrilotriacetic acid chelation moiety and loaded with divalent nickel ions (Ni-NTA) used as a matrix for the selection step. After a counter-selection step using empty Ni-NTA agarose beads, 10 DNA and RNA SELEX cycles were accomplished in parallel (Figure 1) at 37 °C in selection buffer (SB). Individual DNA and RNA aptamers were cloned and sequenced in each of the two final populations (termed 05DS10 and 05RS10, respectively), rendering 26 DNA and 32 RNA molecular sequences (Supplementary Tables S1 and S2). 

The nucleotide composition of DNA and RNA selected aptamers (Supplementary Figure S2), showed a bias towards pyrimidine nucleotides in both processes (58.8% and 58.2% of the DNA and RNA sequences, respectively) as well as a predominance of T/C or U/C tracks. In turn, no repeated sequences among the 58 individual aptamers were observed, with the exception of the DNA molecules, 05DS10-02 and 05DS10-03. 

### 2.2. (RT)qPCR Calibration Curves

**Figure 2:**
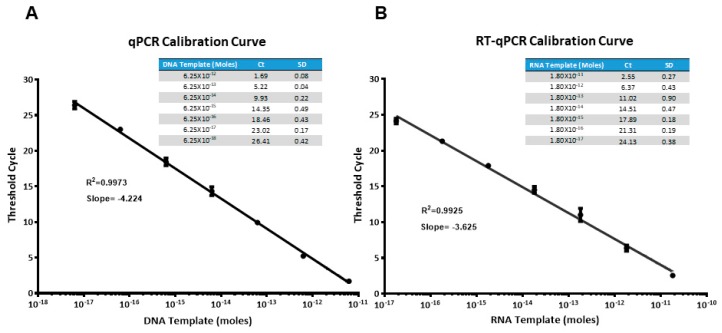
Calibration curves obtained for the amplification of DNA (**A**) and RNA (**B**) templates (molecules of D-ACTG and R-ACUG, respectively) using real time qPCR or RTqPCR, with SYBR green as the fluorophore. Average threshold cycle (*Ct*) of three PCR replicas for each template concentration is shown in the table inset.

Before setting up the ELONA-(RT)qPCR methodology for the affinity assay and further characterization of aptamers, qPCR and RTqPCR calibration curves were constructed, using SYBR green as a fluorophore and the template molecules, D-ACTG and R-ACUG, respectively (see Materials and Methods). They were chosen as non-biased templates to represent the whole set of DNA or RNA aptamer molecules selected in our work, regardless of their sequence composition. As shown in Figure 2, a linear range of seven orders of magnitude (with a correlation coefficient, R^2^, higher than 0.99) was achieved for both DNA and RNA templates. The minimum quantity of template DNA and RNA detected was in the attomolar range: 6.25 × 10^−18^ mol of DNA (corresponding to a *Ct* value of 26.41) and 1.80 × 10^−17^ mol of RNA (*Ct* = 24.13). Then, using these calibration curves, the amount of aptamer bound to PCBP-2 after the partition step of the last SELEX round was quantified by interpolating the average *Ct* value obtained in the ELONA-(RT)qPCR experiments. The possibility that shorter PCR or RT-PCR products could have resulted from skipping some of the ACTG or ACUG repeats of such template molecules was discarded by analysing the amplification products by gel electrophoresis (Supplementary Figure S3).

### 2.3. Affinity Analyses Using ELONA-(RT)qPCR

**Figure 3:**
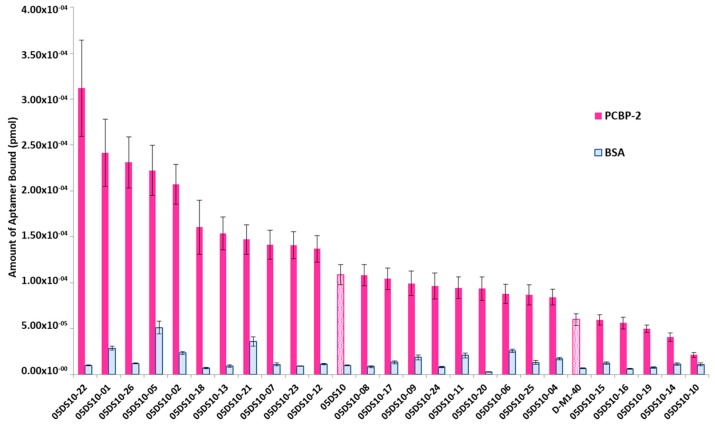
Screening of the PCBP-2 binding affinity of the ssDNA aptamers selected after 10 rounds of SELEX process, using ELONA-qPCR. Recovered aptamers were amplified by qPCR, and the *Ct* values (see text for details) were interpolated within the previously constructed calibration curve (Figure 2A) to quantify the amount of aptamer (pmol) bound to PCBP-2. The mean and standard deviation values of three independent experiments are shown. The whole aptamer population present at the final SELEX round (05DS10) and the random population used as the starting material for the selection process (D-M1-40) were assayed in parallel.

**Figure 4:**
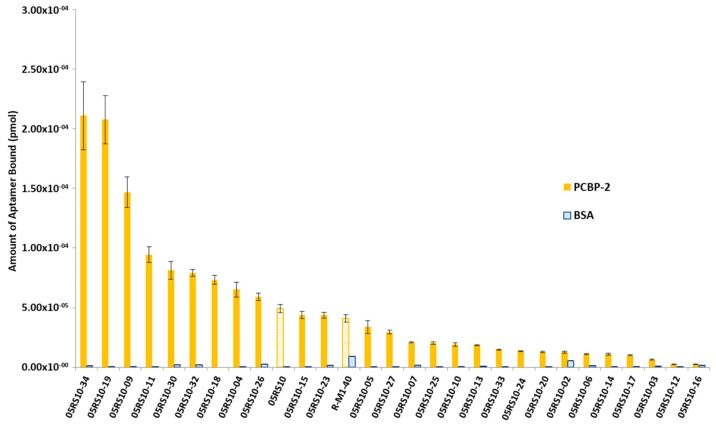
Screening of the PCBP-2 binding affinity of the RNA aptamers selected after 10 rounds of the SELEX process, using ELONA-RTqPCR. The *Ct* values obtained for each experiment (see text and Figure 3 legend for details) were interpolated using the corresponding calibration curve (Figure 2B). The mean and standard deviation (not visible for the BSA data) values of three independent experiments are shown. The whole aptamer population present at the final SELEX round (05RS10) and the random population used as the starting material for the selection process (R-M1-40) were assayed in parallel.

The interest in developing ELONA-(RT)qPCR methodology was twofold. First, it was used to assay, in a 96-well format, the affinity and specificity of the individual selected aptamers. With that purpose, a fixed amount of PCBP-2 (10 pmol), or the same amount of bovine serum albumin (BSA, used as a control of specificity), was immobilized onto Cu^2+^-coated polystyrene plates, further incubated with 10 pmol of each individual DNA or RNA aptamer (Supplementary Figure S1A,C). Such a methodology allowed us to analyse 25 DNA and 27 RNA individual aptamers (Figure 3 and Figure 4). Among them, 11 DNA and 9 RNA aptamers showed a higher binding capacity than both the final population as a whole (05DS10 or 05RS10, respectively) and the initial random pools (D-M1-40 and R-M1-40). All of them showed a high specificity for PCBP-2 with respect to BSA, which was more evident in the case of RNA aptamers (Figure 4). 

### 2.4. Determination of Kd and Bmax Using ELONA-(RT)qPCR 

The second use of ELONA-(RT)qPCR assays was to quantify the binding constants, *Kd* and *Bmax,* of those individual DNA and RNA aptamers showing the highest affinity in the previous assay. Hence, 16 aptamers were chosen among those that revealed a high binding capability and specificity for PCBP-2: 05DS10-01, 05DS10-02, 05DS10-05, 05DS10-12, 05DS10-18, 05DS10-21, 05DS10-22, and 05DS10-26 (DNA aptamers); 05RS10-04, 05RS10-09, 05RS10-11, 05RS10-18, 05RS10-19, 05RS10-30, 05RS10-32, and 05RS10-34 (RNA aptamers). The experiments involved the immobilization of 10 pmol of PCBP-2 to a Cu^2+^-coated polystyrene plate and its incubation with a titration of each aptamer (from 4.06 to 520 nM, Supplementary Figure S1B) in SB. 

**Figure 5:**
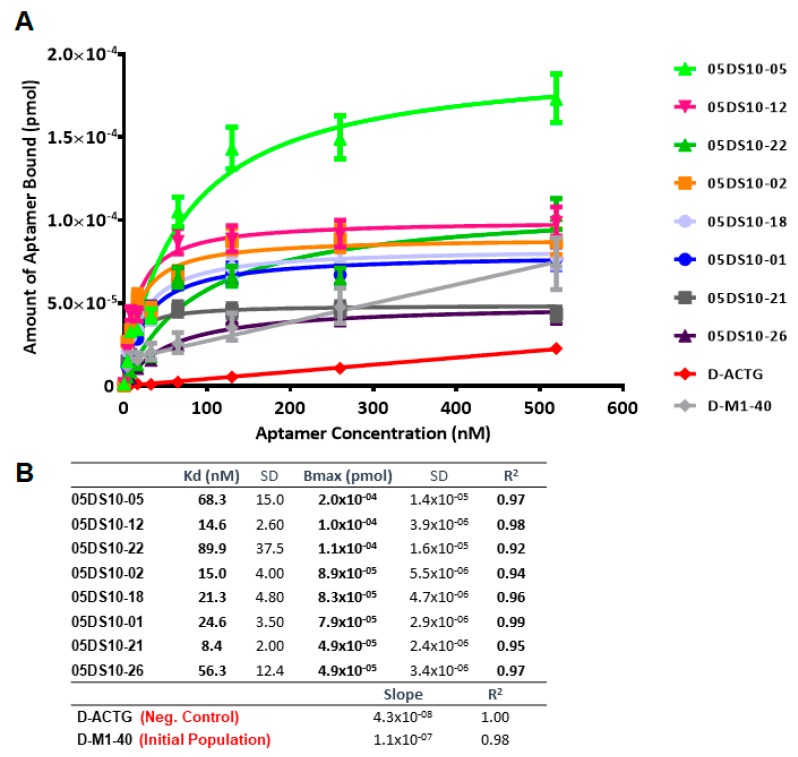
Characterization of the high affinity ssDNA individual aptamers present in the last SELEX round, by means of ELONA-qPCR. The affinity curves obtained for the eight selected individual aptamers (**A**) showed that the best fit curve corresponded to a one site-specific binding model (with R^2^ values in the range of 0.92–0.99) in all cases, from which the *Kd* and *Bmax* values were derived (**B**). In parallel, both the D-ACTG molecule used as a negative control and the initial population of D-M1-40 (see text for details) could only be adjusted to a linear regression model, thus showing non-specific binding to PCBP-2.

**Figure 6:**
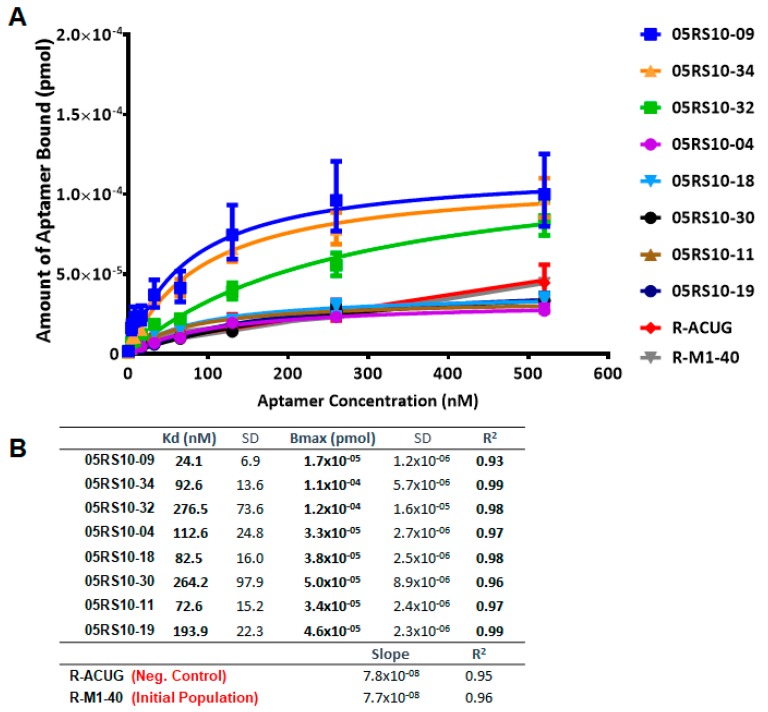
Characterization of the high affinity RNA individual aptamers present in the last SELEX round, using ELONA-RTqPCR. Affinity curves (**A**) and derived *Kd* and *Bmax* values (**B**) of eight selected RNA aptamers and their corresponding controls (R-ACUG and R-M1-40, see text and Figure 5 legend for details). The upper limit of the *y* axis in panel A was chosen to be the same as that in Figure 5, to allow a direct comparison of the performance of ssDNA and RNA aptamers targeting PCBP-2.

The affinity curves obtained using the ELONA-(RT)qPCR approach (Figure 5 and Figure 6) showed that the best fit curve corresponded to a one site-specific binding model (with R^2^ values in the range of 0.92–0.99) in all cases, from which the values of *Kd* and *Bmax* were computed. In parallel, the molecules used as negative controls (D-ACTG and R-ACUG) and the initial populations (D-M1-40 and R-M1-40) could only be adjusted to a linear regression model, thus revealing unspecific binding to PCBP-2. All the assayed aptamers showed *Kd* values in the nanomolar range: From 8.4 to 89.9 nM for DNA aptamers and from 24.1 to 276.5 nM for RNA aptamers. In turn, the *Bmax* values were in the range of 4.9 × 10^−5^ to 2.0 × 10^−5^ pmol (DNA) and 1.7 × 10^−5^ to 1.2 × 10^−4^ pmol (RNA). Thus, the highest affinity for PCBP-2 was achieved by a DNA aptamer (05DS10-21, showing *Kd* = 8.4 nM), while the fittest RNA aptamer showed an affinity three times smaller (05RS10-09, with *Kd* = 24.1 nM). Regarding the maximum binding capacity, the DNA selection process produced the best aptamer (05DS10-05, showing *Bmax* = 2.0 × 10^−4^ pmol), almost two times higher than the RNA aptamer endowed with the maximum PCBP-2 binding (05RS10-32, with *Bmax* = 1.2 × 10^−4^ pmol).

We investigated whether the qPCR and RTqPCR calibration curves used could differ when some of these DNA or RNA aptamer sequences were amplified instead of the D-ACTG and R-ACUG templates. The obtained calibration curves for the different DNA and RNA templates overlapped along a linear range of six orders of magnitude (Supplementary Figure S4). This result discards a relevant effect of the relative amplification efficiencies of individual DNA or RNA sequences. Consequently, it justifies the use of D-ACTG and R-ACUG as non-biased sequences for screening the whole set of DNA or RNA aptamer molecules selected in our work.

### 2.5. Secondary Structure Prediction of the High Affinity DNA and RNA Aptamers

The predicted secondary structure of the 16 aptamers was calculated using Mfold software [12] for DNA aptamers (Supplementary Figure S5) or the Vienna RNA package [11] for RNA aptamers (Supplementary Figure S6). Hairpin or bulge loops containing three or more consecutive pyrimidines were detected in the variable region of four DNA aptamers (05DS10-12, 05DS10-18, 05DS10-21, and 05DS10-22), as well as in six RNA aptamers (05RS10-09, 05RS10-11, 05RS10-18, 05RS10-19, 05RS10-30, and 05RS10-32). Interestingly, the fittest RNA aptamer (05RS10-09) showed a 12 nt-long bulge containing five consecutive pyrimidines (5′-UUCUU-3′). Other high affinity RNA aptamers (05RS10-11, 05RS10-30, and 05RS10-32) also showed 5-6 pyrimidine tracks in internal or hairpin loops (5′-CUUCC-3′, 5′-UUUCU-3′, and 5′-UUUUCU-3′, respectively). These findings complement previous results showing that the 5′-UCCCU-3′ pentanucleotide was the preferred RNA binding sequence to PCBP-2 [24,25]. However, a correlation between the secondary structure of the selected aptamers and their binding capacity was not obvious, and will be the subject of further studies in our group. Among the 16 aptamers assayed, the highest predicted folding stability was shown by 05RS10-30, with a minimum free energy (MFE) of –23.40 kcal/mol (Supplementary Figure S6).

### 2.6. Colorimetric ELONA as an Alternative Technique to Analyse Selected DNA Aptamers

Colorimetric ELONA was set up to assess the overall reliability of the *Kd* values of high affinity DNA aptamers. The obtained *Kd* values of the digoxigenin-labelled version of the eight DNA aptamers previously analysed using ELONA-qPCR ranged from 9.9 to 109.5 nM (Supplementary Figure S7), a nanomolar range similar to that previously obtained using ELONA-qPCR. However, the behaviour of the individual aptamers (with the exception of 05DS10-02) was quantitatively different in both readouts, likely due to the experimental variables involved in qPCR amplification and colorimetric detection. 

### 2.7. Binding of RNA Aptamers to PCBP-2 in Solution

**Figure 7:**
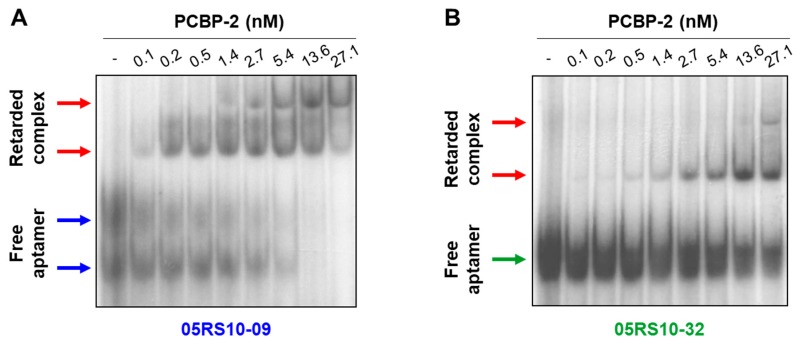
EMSA of the aptamer–target complexes formed by two RNA aptamers showing high affinity for PCBP-2: 05RS10-09 (**A**) and 05RS10-32 (**B**). The uncropped gel images, using short and long exposure times, are shown in Supplementary Figure S8.

EMSA was performed to test, in different experimental conditions, the binding capability of some RNA aptamers previously analysed by ELONA-RTqPCR. RNA-protein binding assays were carried out in solution at room temperature, the labelled RNA aptamer concentration was kept constant (1 nM), and PCBP-2 was titrated (concentration range of 0.1 to 27.1 nM). The results (Figure 7 and Supplementary Figure S8) showed a dose-dependent increase of retarded complex formation of the aptamers, 05RS10-09 and 05RS10-32, with an increasing concentration of PCBP-2. Interestingly, two different conformations of the free aptamer, 05RS10-09, were detected (Figure 7A). In addition, two retarded complexes were distinguished for each aptamer–protein combination (Figure 7A,B). In both cases, the formation of a second retarded complex with an increasing concentration of PCBP-2 could be due to the addition of more than one copy of either the RNA aptamer or the target protein to the complex. This possibility will be the subject of further investigation by our group. In summary, these results showed that the selected aptamers provide binding sites for PCBP-2 protein in solution.

## 3. Discussion

Among the diverse set of molecules involved in the regulation of post-transcriptional pathways, poly(C)-binding proteins (PCBPs) are of special interest due to their role in translation initiation of some viral RNAs [26]. In particular, the cap-independent translation initiation of picornavirus RNA genomes is modulated by the assembly of ribonucleoprotein complexes (involving PCBPs and other host factors) on a highly structured RNA element located at the 5′ untranslated region (5′UTR) of the viral genome, termed the internal ribosome entry site (IRES) [27,28,29,30]. PCBP-2 (also known as hnRNP E2 or CP-2), is a member of the cellular heterogeneous nuclear ribonucleoprotein (hnRNP) family, which is located in the cell nucleus and cytoplasm. hnRNPs bind to cellular proteins and RNAs, mediating various biological processes that include transcriptional regulation, mRNA stabilization, translational control, and apoptotic program activation [31,32,33]. PCBP-2 contains a triplet of K homology (KH) RNA-binding domains, found in the protein component of the human heterogeneous nuclear ribonucleoprotein K (hnRNP K) [34]. KH domains have been identified as nucleic acid (RNA and ssDNA) recognition motifs in proteins that perform a wide range of cellular functions, including transcriptional and translational regulation. Interestingly, PCBP-2 binds to a conserved UC-rich region (containing a CCCUCCC motif) in the 3′-UTR of the androgen receptor (AR) mRNA, thus suggesting a role for PCBP-2 in the modulation of AR mRNA stability and/or translation [26,35]. 

In this work, ssDNA and RNA aptamers against PCBP-2 were selected in parallel, using identical experimental conditions and selection pressures (Figure 1). The lack of convergence found towards a limited number of aptamer sequences after 10 rounds of in vitro selection could be due, among other possibilities, to the above mentioned fact that PCBP-2 contains a triplet of KH nucleic acid-binding domains [34], which likely correlated with a basal nucleic acid binding capacity along the SELEX processes. 

Therefore, the in vitro DNA and RNA selection processes performed exemplify the need for an experimental approach that allows the screening of the binding affinity and specificity of the selected aptamers, beyond their individual sequences or secondary structures. Assays that effectively quantify aptamer-target interactions include colorimetric ELONA [23], flow cytrometry [36], surface plasmon resonance (SPR) [37], isothermal titration calorimetry [38], EMSA [39], or capillary electrophoresis [40]. However, each assay shows some benefits and drawbacks, its suitability depending on both the target features and the type of aptamers (i.e., DNA or RNA) selected [13,14,15]. Additionally, most of these techniques are laborious and/or time consuming, others are not cost effective, and some of them require labelling of either the target or the aptamer. Conversely, these methodologies are not suitable for the analysis of a large number of DNA and RNA aptamers simultaneously. To overcome such technical hitches, we agreed to make use of ELONA as a highly specific and potentially high-throughput assay, in combination with real time PCR (qPCR or RTqPCR) as a quantitative readout instead of the traditional colorimetric methods.

The ELONA-(RT)qPCR methodology here developed allowed us to assay 52 individual DNA and RNA aptamers selected against PCBP-2, as well as to further analyse the 16 fittest ones, avoiding the need for either target/aptamer labelling or secondary recognition reaction. Additionally, (RT)qPCR amplification (using SYBR green as the fluorophore instead of specific fluorescent probes) provided sensitive detection of both DNA and RNA aptamers (down to 6 attomol, Figure 2). A somewhat similar qPCR-based readout was reported by Pinto et al. [41] to detect thrombin using DNA aptamers in a sandwich format. These authors also applied their so called ‘apta-PCR’ to the detection of other targets [42,43]. However, such an assay was not used to comparatively analyse a large number of individual DNA aptamers. Furthermore, RNA aptamers had not been previously assayed using RTqPCR-based methods.

Our method allows a mid-range sample throughput (up to 16 DNA and/or RNA aptamers can be screened in triplicate in a 96-well plate), fast (the complete protocol lasts less than 4 h), and cost effective (thanks to the use of standard well plates and conventional qPCR with SYBR green, avoiding aptamer or target labelling) analysis of individual aptamers. The *Kd* and *Bmax* values of the selected aptamers were quantified for RNA and DNA aptamers in this report (not calculated for DNA aptamers in previous apta-PCR studies [41,42,43]). Additionally, several validation experiments were performed. Firstly, colorimetric ELONA was used to measure the *Kd* values of the eight high affinity DNA aptamers, which remain in the same range as that obtained by ELONA-qPCR. However, quantitative differences detected using both readouts may be due to the experimental variables involved in each of them, including the accessibility of the digoxigenin to the anti-digoxigenin antibody in the colorimetric ELONA, which relies on the (unknown) solution structure of each assayed aptamer. In turn, the RTqPCR-based method developed here allows the screening and analysis of a number of RNA aptamers in parallel, avoiding the need for RNA labelling. Furthermore, the binding of representative in vitro selected RNA aptamers to PCBP-2 in solution was confirmed by EMSA (Figure 7). We have also analysed, in a preliminary study using atomic force microscopy (AFM) [44], the specific binding of a 5′ thiol-modified version of the DNA aptamer 05DS10-21 to recombinant PCBP-2 that includes a GST-tag at its N-terminal end (purchased from Abnova, Taiwan). This allowed us to test the performance of a novel biosensor platform based on aptamers bound to thiol-functionalized graphene.

## 4. Materials and Methods

### 4.1. Expression and Purification of PCBP-2

*E. coli* BL21(DE3) cells transformed with a plasmid expressing 6 × His-PCBP-2 [45] grown at 37 °C were induced with 0.5 mM IPTG for 2 h. Bacterial cell lysates were prepared in binding buffer (20 mM NaH_2_PO_4_, 500 mM NaCl, 20 mM imidazole, 5 mM DTT) as described [46]. Cell debris were eliminated by centrifugation at 12,000 g for 30 min at 4 °C. The lysate was loaded in His-GraviTrap columns (HealthCare) and the recombinant protein was eluted using 500 mM imidazol. The mobility of the eluted PCBP-2 was visualized on coomassie blue stained gels after electrophoresis. Proteins were dialyzed against phosphate buffer, pH 6.8, 1 mM DTT, and stored at –20 °C in 50% glycerol.

### 4.2. DNA and RNA Selection Protocol

For each SELEX round (Figure 1), 50 pmol of PCBP-2 were diluted in selection buffer (SB, composed of 100 mM NaCl, 6 mM MgCl_2_, and 100 mM HEPES pH 7.4) and immobilized onto 25 µL Ni-NTA agarose beads (ThermoFisher, Madrid, Spain) following the manufacturer’s guidelines. Such complexes were used as the selection matrix for the SELEX processes. The designed ssDNA library, termed M1-40, contained a pool of 76 nucleotides (nt)-long oligonucleotides showing a central randomized region of 40 nt flanked by two conserved, 18 nt-long primer-binding sequences: 5′-GCGGATCCAGACTGGTGT(N_40_)GCCCTAAAGACAAGCTTC-3′. This synthetic random DNA library and the DNA primers used for nucleic acid amplifications were purchased (HPLC-purified) from IBA Lifesciences.

The starting material for the in vitro selection consisted in, roughly, 10 copies of each molecule present in the original M1-40 population. To prepare it, 80 pmol (equivalent to 4.8 × 10^13^ molecules) of M1-40 were amplified by PCR using the forward primer, M1-F (5′-GCGGATCCAGACTGGTGT-3′), and the reverse, 5′-phosphorylated primer M1-R-5′P (5′-GAAGCTTGTCTTTAGGGC-3′). Four cycles of PCR reaction (96 °C for 30 s, 60 °C for 1 min, and 72 °C for 1 min) were performed in a final volume of 1 mL, with 200 µM each dNTP and 0.05 U/µL Expand High Fidelity Polymerase (Roche) [47]. The reverse DNA strand was removed by enzymatic digestion using λ exonuclease (New England Biolabs), through incubation at 37 °C for 30 min followed by the enzyme inactivation at 75 °C for 10 min. PCR and digestion cleanup was achieved using Amicon Ultra-0.5 mL Centrifugal Filters (ThermoFisher).

The first SELEX round was carried out using 800 pmol (equivalent to 4.8 × 10^14^ molecules) of the prepared ssDNA population, resuspended in 90 µL of SB, denatured at 95 °C for 10 min, and renatured at 37 °C for 10 min. The folded ssDNA pool was added to 90 µL of selection matrix (Ni-NTA agarose beads bound to PCBP-2) and incubated at 37 °C for 30 min. In the partition step, unbound ssDNA was removed from the selection matrix by centrifugation at 16,000 g for 2 min and washing twice with 250 µL of SB. Then, ssDNA–PCBP-2–bead complexes were resuspended in 90 µL of Milli-Q water, and ssDNA was denatured and eluted by heating at 95 °C for 5 min. The amplification of eluted ssDNA to start the next SELEX round was performed using 5 to 20 cycles (adjusted in each SELEX round depending on the amount of ssDNA recovered) of assymetric PCR using a 100-fold excess of the forward primer, M1-F, with respect to the reverse primer, M1-R-5′P. The PCR reaction (96 °C for 30 s, 60 °C for 1 min, and 72 °C for 1 min) containing up to 1 pmol of template was performed in a final volume of 50 µL, with 200 µM each dNTP and 0.05 U/µL Expand High Fidelity Polymerase. The residual presence of the reverse DNA strand was removed by enzymatic digestion using λ exonuclease as described above. To foster the isolation of high affinity aptamers, the stringency of the SELEX process was increased by reducing the amount of ssDNA bound to the selection matrix (and, thus the ssDNA:PCBP-2 ratio) from 800 pmol (round 1) to 80 pmol (rounds 2 to 10), as well as by doubling the number of washing steps every two rounds. A counter-selection step (using Ni-NTA agarose beads without any bound protein) was introduced before the first round to discard aptamers that could have been selected against the inert matrix. The complete SELEX method consisted of 10 amplification-selection rounds.

In turn, for the selection of RNA aptamers, 80 pmol (4.8 × 10^13^ molecules) of M1-40 were PCR amplified using the primer, M2-F (5′-*TGTAATACGACTCACTATA*GGGGCGGATCCAGACTGGTGT-3′, which includes the T7 RNA polymerase promoter, in italics) and M1-R, in a 1 mL reaction with 4 cycles. Milli-Q water treated with DEPC was used in all the processes involving RNA. Thereafter, the amplified product was in vitro transcribed (IVT) by T7 RNA polymerase (using the AmpliScribe™ T7 High Yield Transcription Kit, Epicentre). The resulting RNA pool was purified by ethanol precipitation. As in the case of ssDNA aptamers, the first SELEX round was performed using 800 pmol of RNA population renatured in 90 µL of SB. After incubation with 90 µL of selection matrix at 37 °C for 30 min, the unbound RNA was removed by centrifugation and washing. RNA–PCBP-2–beads complexes were resuspended and RNA was eluted as detailed above. The recovered aptamers were retro-transcribed and amplified by one-step RT-PCR (with SuperScript^®^ III and Platinum^®^ *Taq* DNA Polymerase, ThermoFisher) using M2-F and M1-R in a total volume of 100 µL (8 to 15 PCR cycles were used in each SELEX round, depending on the amount of RNA recovered), followed by in vitro transcription of 2 µg of the amplified DNA pool. Other experimental variables were the same as those described for the ssDNA process.

### 4.3. Molecular Cloning and Sequencing of Individual Aptamers

The individual aptamers present at the last round of DNA or RNA SELEX processes were cloned. With that aim, the products of the PCR-amplified DNA (or RT-PCR-amplified RNA) were cloned using the TOPO TA cloning PCR II Kit (ThermoFisher) and the recombinant plasmids were used to transform One Shot Mach1-T1 (ThermoFisher) competent *E. coli* strain. Then, 26 DNA and 32 RNA individual aptamers were sequenced by capillary electrophoresis using an ABI 3730xl DNA Analyzer (ThermoFisher) and following standard protocols.

### 4.4. Real Time qPCR and RTqPCR

All reactions were carried out in a StepOnePlus Real-Time PCR System (ThermoFisher): qPCRs were performed using KAPA SYBR FAST qPCR Master Mix (Sigma, Madrid, Spain), whereas RTqPCRs were carried out with SuperScript™ III Platinum™ One-Step qRT-PCR Kit (ThermoFisher). Both kits contained SYBR Green I dye (Sigma). The final volume of all the reactions was 10 µL, containing 4.7 µL of the DNA or RNA template. For the construction of the calibration curves, serial dilutions of the template molecules of D-ACTG and R-ACUG (Figure 2) were used, ranging from 6.25 × 10^−12^ to 6.25 × 10^−18^ mol in DNA and 1.8 × 10^−11^ to 1.8 × 10^−17^ mol in RNA.

### 4.5. ELONA-(RT)qPCR for Affinity Analysis

ELONA-(RT)qPCR experiments were performed by attaching 10 pmol of PCBP-2 to three contiguous wells of a Greiner Bio-One^TM^ 96-Well High Binding Standard ELISA microplate (ThermoFisher) while three additional wells were used to attach the same amount of BSA as a control of the specificity (Supplementary Figure S1A). The proteins were incubated (at 37 °C for 60 min, in SB) with 10 pmol of each individual ssDNA or RNA aptamer (100 nM concentration in the final reaction volume of 100 µL). After three washing steps with SB to remove unspecific binding, PCBP-2 or BSA-bound aptamers were denatured and eluted with water at 95 °C. Recovered aptamers were then amplified by qPCR or RTqPCR in triplicate, and the retrieved *Ct* values (average of the 9 values obtained for each ELONA experiment) were interpolated within the corresponding, previously constructed calibration curve (Figure 2) to quantify the amount of aptamer bound to PCBP-2. In parallel with the individual aptamers assayed, the whole aptamer population of the last SELEX rounds (termed 05DS10 for the ssDNA SELEX and 05RS10 for the RNA process) as well as the initial random pool (M1-40) were subject to ELONA-(RT)qPCR in control experiments.

### 4.6. ELONA-(RT)qPCR for the Functional Characterization of Aptamers

The individual aptamers showing binding ability to PCBP-2 higher than that of the last SELEX round population and M1-40 starting library were subject to further analysis by means of ELONA-(RT)qPCR. For that, PCBP-2 (10 pmol) was attached to each of the 96 wells of a Cu^2+^-coated, high capacity 96-well plate and it was incubated (in triplicate) with eight increasing concentrations (4.06, 8.13, 16.25, 32.50, 65, 130, 260, and 520 nM) of each individual aptamer. Therefore, four different aptamers could be assayed in parallel in a single plate (Supplementary Figure S1B). All the ELONA experiments were repeated in two independent plates, thus 6 wells were used for assaying each aptamer concentration. After 60 min of incubation in SB at 37 °C and three washing steps in SB, PCBP-2-bound aptamers (Supplementary Figure S1C) were denatured and eluted with ultra-pure water at 95 °C. Recovered aptamers were amplified by (RT)qPCR. The average *Ct* values derived from two independent amplifications (corresponding to 12 *Ct* values for each aptamer concentration) were interpolated within the previously constructed calibration curves (Figure 2) to quantify the amount of bound aptamer (pmol) corresponding to each (DNA or RNA) aptamer concentration initially incubated in the ELONA.

In parallel, two molecules were designed and assayed as negative controls in the ELONA-(RT)qPCR experiments, whose sequences contained 10 repeats of either ACTG (ssDNA aptamers) or ACUG (RNA aptamers) tetranucleotides in their central regions. They were termed D-ACTG (5′-GCGGATCCAGACTGGTGT(ACTG)_10_GCCCTAAAGACAAGCTTC-3′) and R-ACUG (5′-GGGGCGGAUCCAGACUGGUGU(ACUG)_10_GCCCUAAAGACAAGCUUC-3′), respectively. Additionally, the initial random population of D-M1-40 (and its RNA version, R-M1-40: 5′-GGGGCGGAUCCAGACUGGUGU(N_40_)GCCCUAAAGACAAGCUUC-3′) was also used as a reference. All the affinity curves obtained were adjusted to alternative binding models using GraphPad Prism software, and those showing higher R^2^ values were selected to calculate the corresponding *Kd* and *Bmax* parameters for each individual DNA or RNA aptamer.

### 4.7. Colorimetric ELONA for the Functional Characterization of ssDNA Aptamers

The eight previously assayed ssDNA aptamers, as well as the D-ACTG negative control, were subject to further analysis by means of colorimetric ELONA. With that aim, PCBP-2 (10 pmol) was attached to a Cu^2+^-coated, high capacity 96-well plate (Supplementary Figure S1B) and it was incubated (in triplicate) with eight increasing concentrations (3.91, 7.81, 15.63, 31.25, 62.50, 125, 250, and 500 nM) of each individual, 3′ digoxigenin-labelled ssDNA aptamer (IBA Lifesciences). All the ELONA experiments were repeated in two independent plates, thus 6 wells were used for assaying each aptamer concentration. After a 60 min incubation in SB at 37 °C and three washing steps in SB, PCBP-2-bound aptamers were incubated for 60 min in SB with 100 μL anti-digoxigenin antibody diluted 1:5000 and labelled with HRP (Roche), following the manufacturer’s instructions. The reaction mixture was washed three times in SB, and a colorimetric reaction was developed by adding 100 μL of 3,3′,5,5′-tetramethylbenzidine (TMB). After incubating the reaction for 10 min, it was stopped by adding 100 μL of 2 M sulphuric acid. Absorbance at 450 nm was recorded and plotted to quantify the amount of ssDNA aptamer bound to PCBP-2 that corresponds to each aptamer concentration initially incubated in the ELONA. Then, *Kd* and *Bmax* values were calculated as explained above.

### 4.8. Electrophoretic Mobility Shift Assays (EMSA) of Aptamer–PCBP-2 Complexes

RNA aptamer synthesis was performed by in vitro transcription using T7 RNA polymerase. With that purpose, the dsDNA template version of the RNA aptamers (0.5 µg/µL) was transcribed in 40 mM Tris-HCl pH 7.9, 6 mM MgCl_2_, 50 mM DTT, 0.5 mM rNTPs (final volume 10 µL) at 37 °C for 2 h. Transcripts were uniformly labelled using α^32^P-CTP (500 Ci/mmol) and RNA was separated from non-incorporated isotope using G-25 (HealthCare) spin columns. Labelled RNA was extracted with phenol-chloroform, ethanol precipitated, and resuspended in TE to a concentration of 0.04 pmol/µL. RNA integrity was examined in 6% acrylamide 7 M urea denaturing gel electrophoresis, as described [48]. Next, aptamer–PCBP-2 binding reactions were carried out at room temperature for 20 min, in 10 μL of binding buffer (40 mM Tris-HCl pH 7.5, 250 mM NaCl, 0.1 % (*w*/*v*) βME). Increasing amounts of protein (0.1 to 27.1 nM) were incubated with a fixed concentration of ^32^P-labelled RNA (about 10^3^ cpm, 0.01 pmol) for 20 min at room temperature, as described [49]. Following the addition of xylene cyanol buffer (10 mM Tris-HCl pH7.5, 48.5% Glycerol, 0.5% xylene cyanol), the samples were subjected to non-denaturing 8.0% (29:1) acrylamide/bis-acrylamide gel electrophoresis. The gels were run at 100 V for 4 h in 1X TBE buffer (90 mM Tris-HCl pH 7.5, 64.6 mM boric acid, 2.5 mM EDTA, pH 8.4) at 4 °C. The ^32^P-labelled RNA and retarded aptamer–PCBP-2 complexes were detected by autoradiography (for 16 h and 80 h, at –80°C) of dried gels.

## 5. Conclusions

Here, we report the development and characterization of the first described DNA and RNA aptamers against the protein, PCBP-2. From a technological point of view, we demonstrated the usefulness and general applicability of the ELONA-(RT)qPCR approach for the functional analysis of DNA and, noticeably, RNA aptamers. This methodology allows the quantification of their kinetic constants, *Kd* and *Bmax*, in a label-free and cost-effective format. Therefore, we envisage the ELONA-(RT)qPCR approach as a general strategy for aptamer characterization, with a broad applicability in the field of aptamer-based biosensors useful in biotechnology and biomedicine.

## Supplementary Materials

The following are available online.

## References

[1] Ellington A.D., Szostak J.W. (1990). In vitro selection of RNA molecules that bind specific ligands. Nature.

[2] Tuerk C., Gold L. (1990). Systematic evolution of ligands by exponential enrichment: RNA ligands to bacteriophage t4 DNA polymerase. Science.

[3] Yuce M., Ullah N., Budak H. (2015). Trends in aptamer selection methods and applications. Analyst.

[4] Gold L., Walker J. (2017). Directed evolution ☆. Reference Module in Life Sciences.

[5] Blank M., Mayer G. (2016). Next-generation analysis of deep sequencing data: Bringing light into the black box of selex experiments. Nucleic Acid Aptamers.

[6] Alam K.K., Chang J.L., Burke D.H. (2015). Fastaptamer: A bioinformatic toolkit for high-throughput sequence analysis of combinatorial selections. Mol. Ther. Nucleic Acids.

[7] Li W., Cowley A., Uludag M., Gur T., McWilliam H., Squizzato S., Park Y.M., Buso N., Lopez R. (2015). The embl-ebi bioinformatics web and programmatic tools framework. Nucleic Acids Res..

[8] Larkin M.A., Blackshields G., Brown N.P., Chenna R., McGettigan P.A., McWilliam H., Valentin F., Wallace I.M., Wilm A., Lopez R. (2007). Clustal w and clustal x version 2.0. Bioinformatics.

[9] Hoinka J., Berezhnoy A., Sauna Z.E., Gilboa E., Przytycka T.M. (2014). Aptacluster—A method to cluster ht-selex aptamer pools and lessons from its application. Res. Comput. Mol. Biol..

[10] Hoinka J., Dao P., Przytycka T.M. (2015). Aptagui[mdash]a graphical user interface for the efficient analysis of ht-selex data. Mol. Ther. Nucleic Acids.

[11] Lorenz R., Bernhart S.H., Höner zu Siederdissen C., Tafer H., Flamm C., Stadler P.F., Hofacker I.L. (2011). Viennarna package 2.0. Algorithms Mol. Biol..

[12] Zuker M. (2003). Mfold web server for nucleic acid folding and hybridization prediction. Nucleic Acids Res..

[13] Sharma T.K., Bruno J.G., Dhiman A. (2017). Abcs of DNA aptamer and related assay development. Biotechnol. Adv..

[14] Tan S.Y., Acquah C., Sidhu A., Ongkudon C.M., Yon L.S., Danquah M.K. (2016). Selex modifications and bioanalytical techniques for aptamer-target binding characterization. Crit. Rev. Anal. Chem..

[15] Ruscito A., DeRosa M.C. (2016). Small-molecule binding aptamers: Selection strategies, characterization, and applications. Front. Chem..

[16] Drolet D.W., Moon-McDermott L., Romig T.S. (1996). An enzyme-linked oligonucleotide assay. Nat. Biotechnol..

[17] Ljungstrom I., Engvall E., Ruitenberg E.J. (1974). Proceedings: Elisa, enzyme linked immunosorbent assay—A new technique for sero-diagnosis of trichinosis. Parasitology.

[18] Vivekananda J., Kiel J.L. (2006). Anti-francisella tularensis DNA aptamers detect tularemia antigen from different subspecies by aptamer-linked immobilized sorbent assay. Lab. Investig..

[19] Park J.H., Jee M.H., Kwon O.S., Keum S.J., Jang S.K. (2013). Infectivity of hepatitis c virus correlates with the amount of envelope protein e2: Development of a new aptamer-based assay system suitable for measuring the infectious titer of hcv. Virology.

[20] Farrell R.E., Farrell R.E. (2010). Chapter 12—Nucleic acid probe technology. RNA Methodologies.

[21] Pauff S., Withers J.M., McKean I.J.W., Mackay S.P., Burley G.A. (2017). Synthetic biological approaches for RNA labelling and imaging: Design principles and future opportunities. Curr. Opin. Biotechnol..

[22] Sive H.L., Grainger R.M., Harland R.M. (2007). Synthesis and purification of digoxigenin-labeled RNA probes for in situ hybridization. CSH Protoc..

[23] Guerra-Perez N., Ramos E., Garcia-Hernandez M., Pinto C., Soto M., Martin M.E., Gonzalez V.M. (2015). Molecular and functional characterization of ssdna aptamers that specifically bind leishmania infantum pabp. PLoS ONE.

[24] Thisted T., Lyakhov D.L., Liebhaber S.A. (2001). Optimized RNA targets of two closely related triple kh domain proteins, heterogeneous nuclear ribonucleoprotein k and ±cp-2kl, suggest distinct modes of RNA recognition. J. Biol. Chem..

[25] Flynn R.A., Martin L., Spitale R.C., Do B.T., Sagan S.M., Zarnegar B., Qu K., Khavari P.A., Quake S.R., Sarnow P. (2015). Dissecting noncoding and pathogen RNA-protein interactomes. RNA.

[26] Martinez-Salas E., Francisco-Velilla R., Fernandez-Chamorro J., Lozano G., Diaz-Toledano R. (2015). Picornavirus ires elements: RNA structure and host protein interactions. Virus Res..

[27] Fujimura K., Kano F., Murata M. (2008). Identification of pcbp2, a facilitator of ires-mediated translation, as a novel constituent of stress granules and processing bodies. RNA.

[28] Perera R., Daijogo S., Walter B.L., Nguyen J.H.C., Semler B.L. (2007). Cellular protein modification by poliovirus: The two faces of poly(rc)-binding protein. J. Virol..

[29] Lozano G., Fernandez N., Martinez-Salas E. (2016). Modeling three-dimensional structural motifs of viral ires. J. Mol. Biol..

[30] Fernandez-Chamorro J., Francisco-Velilla R., Ramajo J., Martinez-Salas E. (2019). Rab1b and arf5 are novel RNA-binding proteins involved in fmdv ires-driven RNA localization. Life Sci. Alliance.

[31] Venables J.P., Koh C.S., Froehlich U., Lapointe E., Couture S., Inkel L., Bramard A., Paquet E.R., Watier V., Durand M. (2008). Multiple and specific mrna processing targets for the major human hnrnp proteins. Mol. Cell. Biol..

[32] Makeyev A.V., Liebhaber S.A. (2002). The poly(c)-binding proteins: A multiplicity of functions and a search for mechanisms. RNA.

[33] Li F., Bullough K.Z., Vashisht A.A., Wohlschlegel J.A., Philpott C.C. (2016). Poly(rc)-binding protein 2 regulates hippo signaling to control growth in breast epithelial cells. Mol. Cell. Biol..

[34] Adinolfi S., Bagni C., Castiglione Morelli M.A., Fraternali F., Musco G., Pastore A. (1999). Novel RNA-binding motif: The kh module. Biopolymers.

[35] Yeap B.B., Voon D.C., Vivian J.P., McCulloch R.K., Thomson A.M., Giles K.M., Czyzyk-Krzeska M.F., Furneaux H., Wilce M.C.J., Wilce J.A. (2002). Novel binding of hur and poly(c)-binding protein to a conserved uc-rich motif within the 3â€²-untranslated region of the androgen receptor messenger RNA. J. Biol. Chem..

[36] Nabavinia M.S., Charbgoo F., Alibolandi M., Mosaffa F., Gholoobi A., Ramezani M., Abnous K. (2018). Comparison of flow cytometry and elasa for screening of proper candidate aptamer in cell-selex pool. Appl. Biochem. Biotechnol..

[37] Chang A.L., McKeague M., Smolke C.D. (2014). Facile characterization of aptamer kinetic and equilibrium binding properties using surface plasmon resonance. Methods Enzymol..

[38] Potty A.S., Kourentzi K., Fang H., Jackson G.W., Zhang X., Legge G.B., Willson R.C. (2009). Biophysical characterization of DNA aptamer interactions with vascular endothelial growth factor. Biopolymers.

[39] Sánchez-Luque F.J., Stich M., Manrubia S., Briones C., Berzal-Herranz A. (2014). Efficient hiv-1 inhibition by a 16 nt-long RNA aptamer designed by combining in vitro selection and in silico optimisation strategies. Sci. Rep..

[40] Stuart C.H., Riley K.R., Boyacioglu O., Herpai D.M., Debinski W., Qasem S., Marini F.C., Colyer C.L., Gmeiner W.H. (2016). Selection of a novel aptamer against vitronectin using capillary electrophoresis and next generation sequencing. Mol. Ther. Nucleic Acids.

[41] Pinto A., Bermudo Redondo M.C., Ozalp V.C., O’Sullivan C.K. (2009). Real-time apta-pcr for 20 000-fold improvement in detection limit. Mol. BioSyst..

[42] Civit L., Pinto A., Rodrigues-Correia A., Heckel A., O’Sullivan C.K., Mayer G. (2016). Sensitive detection of cancer cells using light-mediated apta-pcr. Methods.

[43] Pinto A., Polo P., Henry O., Redondo M.C., Svobodova M., O’Sullivan C. (2014). Label-free detection of gliadin food allergen mediated by real-time apta-pcr. Anal. Bioanal. Chem..

[44] Bueno R., Marciello M., Moreno M., Sánchez-Sánchez C., Martinez J.I., Martinez L., Prats-Alfonso E., Guimerà-Brunet A., Garrido J.A., Villa R. (2019). Versatile graphene-based platform for robust nanobiohybrid interfaces. ACS Omega.

[45] Sean P., Nguyen J.H., Semler B.L. (2009). Altered interactions between stem-loop iv within the 5′ noncoding region of coxsackievirus RNA and poly(rc) binding protein 2: Effects on ires-mediated translation and viral infectivity. Virology.

[46] Francisco-Velilla R., Fernandez-Chamorro J., Lozano G., Diaz-Toledano R., Martinez-Salas E. (2015). RNA-protein interaction methods to study viral ires elements. Methods.

[47] Hall B., Micheletti J.M., Satya P., Ogle K., Pollard J., Ellington A.D. (2001). Design, Synthesis, and Amplification of DNA Pools for in Vitro Selection.

[48] Fernandez N., Garcia-Sacristan A., Ramajo J., Briones C., Martinez-Salas E. (2011). Structural analysis provides insights into the modular organization of picornavirus ires. Virology.

[49] Fernandez-Chamorro J., Pineiro D., Gordon J.M., Ramajo J., Francisco-Velilla R., Macias M.J., Martinez-Salas E. (2014). Identification of novel non-canonical RNA-binding sites in gemin5 involved in internal initiation of translation. Nucleic Acids Res..

