# Supplementary material for: A Combined ELONA-(RT)qPCR Approach for Characterizing DNA and RNA Aptamers Selected against PCBP-2
Source: Molecules. 2019 Mar 28;24(7):1213. doi: 10.3390/molecules24071213 (PMC6480920; doi:10.3390/molecules24071213)
Supplement: Supplementary file 1 [file molecules-24-01213-s001.pdf]

Supplementary Material

A combined ELONA-(RT)qPCR approach for characterizing DNA and RNA aptamers selected against PCBP-2

Miguel Moreno[1], María Fernández-Algar[1], Javier Fernández-Chamorro[2], Jorge Ramajo[2], Encarnación Martínez-Salas[2], Carlos Briones[1,3,*]

[1] *Laboratory of Molecular Evolution. Centro de Astrobiología (CSIC-INTA), Torrejón de Ardoz, Madrid, Spain.*
[2] *Centro de Biología Molecular "Severo Ochoa" (CSIC-UAM), Madrid, Spain.*
[3] *Centro de Investigación Biomédica en Red de Enfermedades Hepáticas y Digestivas (CIBERehd), Spain.*
**Correspondence and requests for materials should be addressed to C.B. (e-mail: cbriones@cab.inta-csic.es)*

SUPPLEMENTARY TABLES

Supplementary Table S1. Sequences of the 26 individual (75 to 76 nt-long) ssDNA aptamers selected after 10 rounds of SELEX process using PCBP-2 as the target molecule. Aptamers marked with an asterisk showed binding capacity to PCBP-2 higher than that of the last SELEX round population and M1-40 starting library (Figure 3), and were further characterized by ELONA-qPCR (Figure 5 and Supplementary Figure S5) as well as colorimetric ELONA (Supplementary Figures S9). Aptamers 05DS10-02 and 05DS10-03 showed identical sequence. Nucleotides highlighted in bold correspond to the 40 nt-long selected sequence within each aptamer.

Name	DNA aptamer sequence
05DS10-01*	GCGGATCCAGACTGGTGT**GCACAGCACATGCATTACTGCGGTAATCCGTGTCCTTTGT**GCCCTAAAGACAAGCTTC
05DS10-02*	GCGGATCCAGACTGGTGT**ACTGGGCTAGTTGCCTCTGCAGATTAGATGATGACAGTG**GCCCTAAAGACAAGCTTC
05DS10-03	GCGGATCCAGACTGGTGT**ACTGGGCTAGTTGCCTCTGCAGATTAGATGATGACAGTG**GCCCTAAAGACAAGCTTC
05DS10-04	GCGGATCCAGACTGGTGT**GGCACTCTTGCTACCGGGACTCTCCCACTTTCTCACGGG**GCCCTAAAGACAAGCTTC
05DS10-05*	GCGGATCCAGACTGGTGT**ACCTGTTAGCAAGAGTTTATTATGTAAAGATATCCGTTGG**GCCCTAAAGACAAGCTTC
05DS10-06	GCGGATCCAGACTGGTGT**AGTTCTCCTCTTTAAGATGCTTTATACGGCGTCTTATTAT**GCCCTAAAGACAAGCTTC
05DS10-07	GCGGATCCAGACTGGTGT**TGTGGCTACTATCCCTTGGTTATAAGTCTCATGCTCGCTG**GCCCTAAAGACAAGCTTC
05DS10-08	GCGGATCCAGACTGGTGT**ACCTGATAGGCTGATCTTAGGTGAGGAGGTTACCTGTCGT**GCCCTAAAGACAAGCTTC
05DS10-09	GCGGATCCAGACTGGTGT**GTACTGTGTCGGTCTATTATTACAAAGTACCCCCCCGTAT**GCCCTAAAGACAAGCTTC
05DS10-10	GCGGATCCAGACTGGTGT**TAACCGGATCGCGCCCTCCTCGCTATCCCCCTCCGTCGGT**GCCCTAAAGACAAGCTTC
05DS10-11	GCGGATCCAGACTGGTGT**CCTCAAACAATCCCGATTCAAACAGCCTCTTCCTTAGTGT**GCCCTAAAGACAAGCTTC
05DS10-12*	GCGGATCCAGACTGGTGT**GCAGGTATGCCGGATCATGTCGTGAAAGTATCCATTTCTC**GCCCTAAAGACAAGCTTC
05DS10-13	GCGGATCCAGACTGGTGT**GGCTCACAGAACAGCCTTGAGTTTTATTTCCCTGCCGTTT**GCCCTAAAGACAAGCTTC
05DS10-14	GCGGATCCAGACTGGTGT**ATCCCTACGCATCGTGTCCTCGACAGACTATGGATCAGTC**GCCCTAAAGACAAGCTTC
05DS10-15	GCGGATCCAGACTGGTGT**GGCGCTGCGTCGCTGTTGGTCCCTCTTTGCCTATTGTTGT**GCCCTAAAGACAAGCTTC
05DS10-16	GCGGATCCAGACTGGTGT**GGGGACGGGTTTCTACCTTAATTCCGTTCCTGGTAACTCC**GCCCTAAAGACAAGCTTC
05DS10-17	GCGGATCCAGACTGGTGT**TTCGGTGGGGTGGTTTAGTATCTGATTCTGTCATGTTGTT**GCCCTAAAGACAAGCTTC
05DS10-18*	GCGGATCCAGACTGGTGT**CCTATCTATAATTTTGCAGTCCACGTTTCTCTTGTGTGTG**CCCTAAAGACAAGCTTC
05DS10-19	GCGGATCCAGACTGGTGT**GGCTTTGCTGTATACAAAGTGCTTTGGTCTTTCGGATTGT**GCCCTAAAGACAAGCTTC
05DS10-20	GCGGATCCAGACTGGTGT**GGCGCCCGTTTTCGCTGCTCACTTCGCAGAAGGTCATCCG**GCCCTAAAGACAAGCTTC
05DS10-21*	GCGGATCCAGACTGGTGT**GGAGGTTAGCCGAAACACGTATACGCGTATTTATCCTCGG**GCCCTAAAGACAAGCTTC
05DS10-22*	GCGGATCCAGACTGGTGT**CAATGGTACTCTTCATTGTAGTCGCTTTGTTTATTAGCCG**GCCCTAAAGACAAGCTTC
05DS10-23	GCGGATCCAGACTGGTGT**TGCAGCATCGCGTCACGCGTCTACATTGTTCGTCTCACC**GCCCTAAAGACAAGCTTC
05DS10-24	GCGGATCCAGACTGGTGT**GCCATTACCATGGATCTGTCACCCGCTCTCTCCCCGGGGC**GCCCTAAAGACAAGCTTC
05DS10-25	GCGGATCCAGACTGGTGT**GGATACGTAACTTGCTATTGATTTTGCAATTGTTGATTAT**GCCCTAAAGACAAGCTTC
05DS10-26*	GCGGATCCAGACTGGTGT**GGGAATGTTGTTTATGTATTTGTTCTGAGCTCTACCTTT**GCCCTAAAGACAAGCTTC

Supplementary Table S2. Sequences of the 32 individual (77 to 79 nt-long) RNA aptamers selected after 10 rounds of SELEX process using PCBP-2 as the target molecule. Aptamers marked with an asterisk showed binding capacity to PCBP-2 higher than that of the last SELEX round population and M1-40 starting library (Figure 4) and were characterized by ELONA-RTqPCR (Figure 6 and Supplementary Figure S6). Nucleotides highlighted in bold correspond to the 40 nt-long selected sequence within each aptamer.

Name	RNA aptamer sequence
05RS10-02	GGGGCGGAUCCAGACUGGUGU**CAUAUGAUUGUGUUUAGCGGGAGUACCUUGAUGUUUUGCG**GCCCUAAAGACAAGCUUC
05RS10-03	GGGGCGGAUCCAGACUGGUGU**CUUGUCUAGGCCGGUAAGAUUGGAUGAUAAUUGUUUGG**GCCCUAAAGACAAGCUUC
05RS10-04*	GGGGCGGAUCCAGACUGGUGU**CAUUUAGCAAAAACACUUGUAUAAUUCAAGUCGAUGUUGG**GCCCUAAAGACAAGCUUC
05RS10-05	GGGGCGGAUCCAGAGUGGUGU**GUUGUUAAACGGUGGAUUGGUUUAUUAGUGUUUAGGCG**GCCCUAAAGACAAGCUUC
05RS10-06	GGGGCGGAUCCAGACUGGUGU**CGCCUUUAGUGUACACAAUAUAUCCUUCCUCUGUUGGGCG**GCCCUAAAGACAAGCUUC
05RS10-07	GGGGCGGAUCCAGACUGGUGU**CGGGAACUAUCGGCUUGCGACUAUUUACCUGUGUCAUUGG**GCCCUAAAGACAAGCUUC
05RS10-08	GGGGCGGAUCCAGACUGGUGU**CAUAUGAUUGUGUUUAGCGGGAGUACCUUGAUGUUUUGCG**GCCCUAAAGACAAGCUUC
05RS10-09*	GGGGCGGAUCCAGACUGGUGU**ACACGGUGUUUAGUAGUUUAAUGAAUCUUUUAGUUCUUGG**GCCCUAAAGACAAGCUUC
05RS10-10	GGGGCGGAUCCAGACUGGUGU**UACCAUUAGCCGACGCCCUCUCUCACUUAUGUGUCGCUGG**GCCCUAAAGACAAGCUUC
05RS10-11*	GGGGCGGAUCCAGACUGGUGU**GAUCAUAUUAAUAAGACGCUUCCAGGUACGUCGCUGUUGG**GCCCUAAAGACAAGCUUC
05RS10-12	GGGGCGGAUCCAGACUGGUGU**UCCUCUGACACUUUCAAAACAUUGGCGUACUUCAUUCGUG**GCCCUAAAGACAAGCUUC
05RS10-13	GGGGCGGAUCCAGACUGGUGU**AUUUGGUAGGGCGUAUUAUUUUAAGAAUUUUGUUGCGUGG**GCCCUAAAGACAAGCUUC
05RS10-14	GGGGCGGAUCCAGACUGGUGU**CAUUAAAAACUAAUCUAUUUCUGGUCGUGUAUAGUCUUGG**GCCCUAAAGACAAGCUUC
05RS10-15	GGGGCGGAUCCAGACUGGUGU**UUUGUUCUAUCGGGUUUCUCAAUGUGUUGUUUGUCAGUGG**GCCCUAAAGACAAGCUUC
05RS10-16	GGGGCGGAUCCAGACUGGUGU**GUUAAUUAAAAACUUUGGUUCCCAUUUUCUCUCUCUUUGG**GCCCUAAAGACAAGCUUC
05RS10-17	GGGGCGGAUCCAGACUGGUGU**UGCGUAAUUUGUGUUUUGAUAUAAGUGUACUCCUCACGCG**GCCCUAAAGACAAGCUUC
05RS10-18*	GGGGCGGAUCCAGACUGGUGU**UUAAUUAUGUAAGUAAAUUGUUUUUUGACUCUCGCAUUGG**GCCCUAAAGACAAGCUUC
05RS10-19*	GGGGCGGAUCCAGACUGGUGU**AAUCGAUCUUGCAUGCUAUUCGUCAAUCAACUCUUGCCGG**CCCUAAAGACAAGCUUC
05RS10-20	GGGGCGGAUCCAGACUGGUGU**UUCCCUAGGACUUCCGACUAGUAAUGUUUGGUUUCCCGUG**GCCCUAAAGACAAGCUUC
05RS10-22	GGGGCGGAUCCAGACUGGUGU**ACUUCUAAAACUUCUCCAGCAGGGAAACUUCGUUCCUUGG**GCCCUAAAGACAAGCUUC
05RS10-23	GGGGCGGAUCCAGACUGGUGU**UCUUUUAAUAUUAUAGGUCUUUUAUUAGUGUGUCUUUGUG**GCCCUAAAGACAAGCUUC
05RS10-24	GGGGCGGAUCCAGACUGGUGU**UCUAACGUCCUAUACUCAAUGGGUAUGCUUGUUUUAUUGG**GCCCUAAAGACAAGCUUC
05RS10-25	GGGGCGGAUCCAGACUGGUGU**UUCCUAUCUUACCCGAGGUAUAACGUUUGAUUCGCGUGG**GCCCUAAAGACAAGCUUC
05RS10-26	GGGGCGGAUCCAGACUGGUGU**CUUUCUGUCCAGCUCUUAGGUUCAUCUUCAGGUCUACUGG**GCCCUAAAGACAAGCUUC
05RS10-27	GGGGCGGAUCCAGACUGGUGU**UGUGGUAUACACUCUGCAUUUUUAUUUUGGACACUCAUGG**GCCCUAAAGACAAGCUUC
05RS10-28	GGGGCGGAUCCAGACUGGUGU**CGAAUAUUAUGAGUGUGUGCCGCAUGUCUUUCCUCGCUCG**GCCCUAAAGACAAGCUUC
05RS10-29	GGGGCGGAUCCAGACUGGUGU**CGUUAUUUAACUUGAUAUUAUUUGAUCAUCGUCACGUUGG**GCCCUAAAGACAAGCUUC
05RS10-30*	GGGGCGGAUCCAGACUGGUGU**UUCCGCAAAGAGUGGUCUUUGUUAAUGUCAGGUUUCUUCG**GCCCUAAAGACAAGCUUC
05RS10-31	GGGGCGGAUCCAGACUGGUGU**UUAAUCCUUACGUCCUUUUGCGGUUUUCGUGUGUUCUUGG**GCCCUAAAGACAAGCUUC
05RS10-32*	GGGGCGGAUCCAGACUGGUGU**CUUCCUGCUUGUGUUAUUUUCUAUUGUCGUGCGUGUUCGG**GCCCUAAAGACAAGCUUC
05RS10-33	GGGGCGGAUCCAGACUGGUGU**GUAGGUGACUUGGUUAUCCUGUUUCACUAACUUUACUUGG**GCCCUAAAGACAAGCUUC
05RS10-34*	GGGGCGGAUCCAGACUGGUGU**GAACACAGACGAGAACGUUGCAUAAAACCGCUUUUUUGG**GCCCUAAAGACAAGCUUC

SUPPLEMENTARY FIGURES

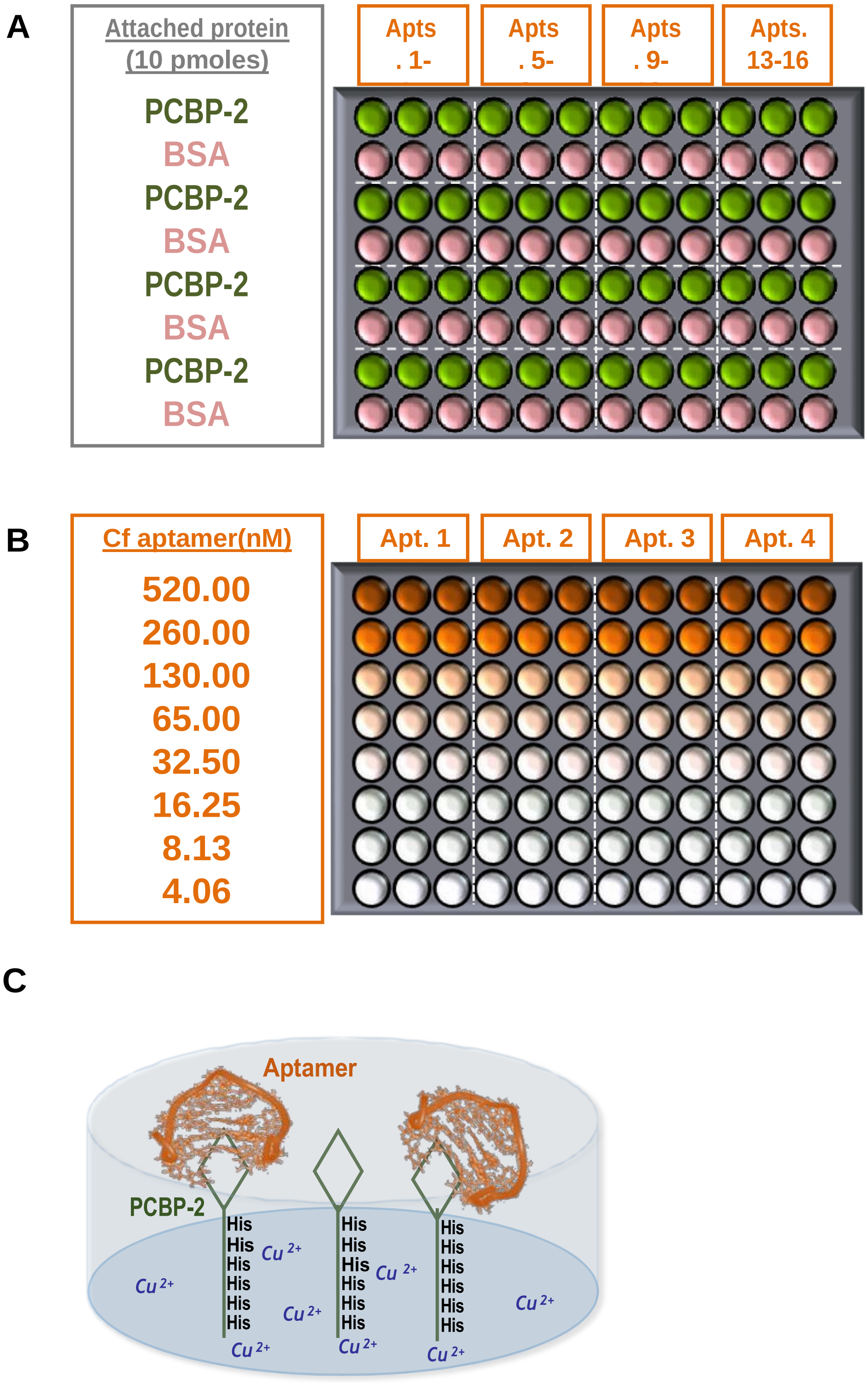

Supplementary Figure S1. Configuration of ELONA-(RT)qPCR experiments performed in high capacity 96-well plates for the affinity analysis (**A**) and the quantification of *Kd* and *Bmax* (**B**) of DNA and RNA aptamers. The schematic representation of aptamer–PCBP-2 binding in the ELONA format corresponding to panel **B** is shown in panel **C**.

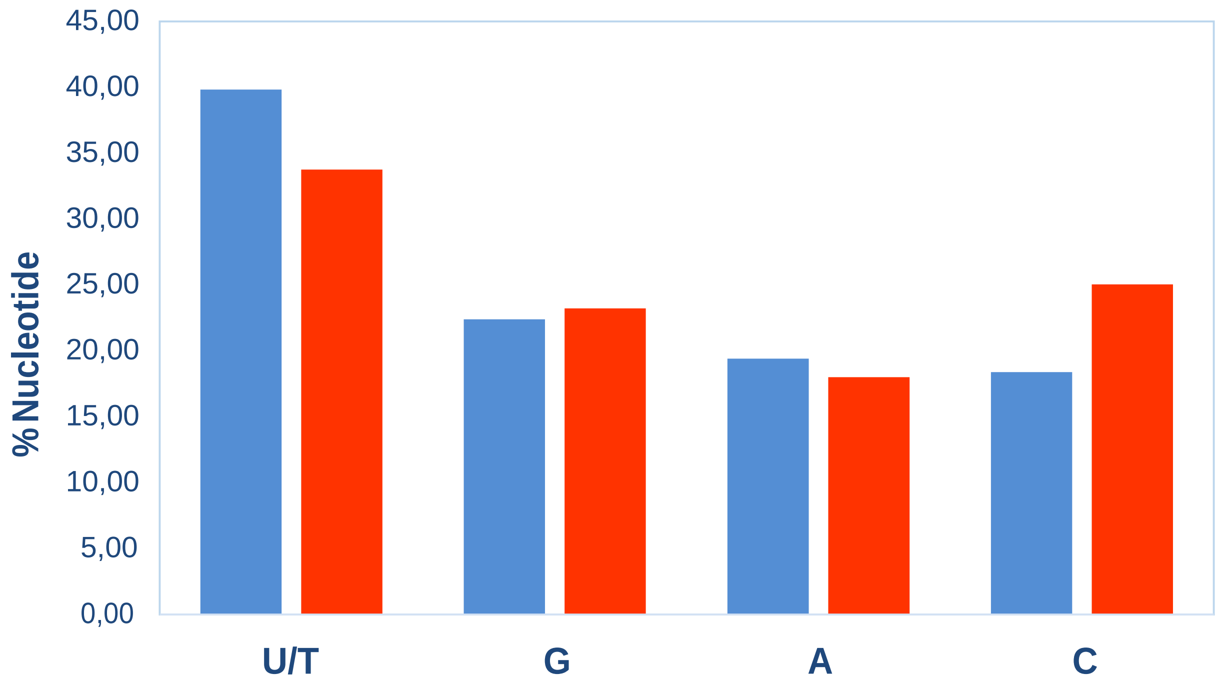

Supplementary Figure S2. Nucleotide composition of the 10th round of RNA (blue bars) and DNA (red bars) aptamers selection against PCBP-2.

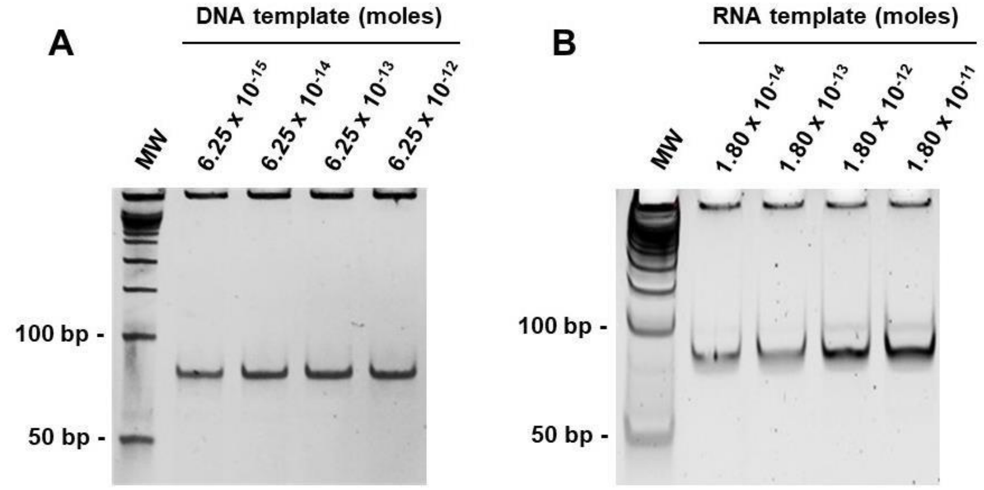

Supplementary Figure S3. Quality control of the amplification products resulting from either qPCR of the template molecule D-ACTG (**A**) or RTqPCR of the template molecule R-ACUG (**B**). The DNA products resulting from (RT)qPCR amplification using the upper template concentrations shown in Figure 2 were loaded in a non-denaturing 10.0% (19:1) acrylamide/bis-acrylamide gel electrophoresis (in 0.5 X TBE), which was run at 100 V for 1 hour. MW: Molecular weight DNA marker (50 pb).

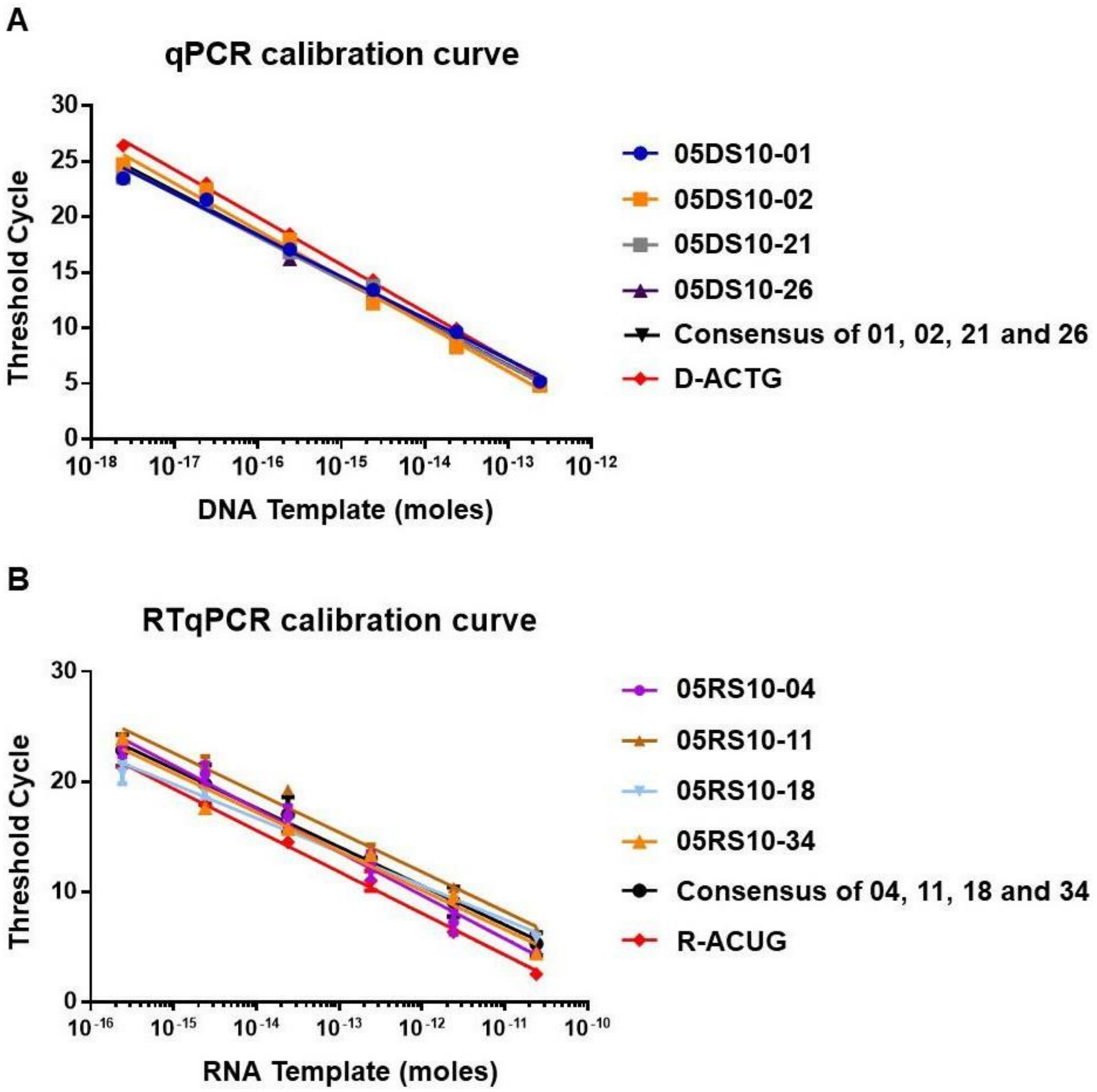

Supplementary Figure S4. Calibration curves obtained using four high affinity DNA (**A**) and RNA (**B**) aptamer molecules as templates for (RT)qPCR amplification, with SYBR Green as fluorophore. Consensus calibration curves for each group of four curves are shown in black, and the curves corresponding to D-ACTG and R-ACUG templates (in red, already depicted in Figure 2) are superimposed in the interval of six orders of magnitude assayed.

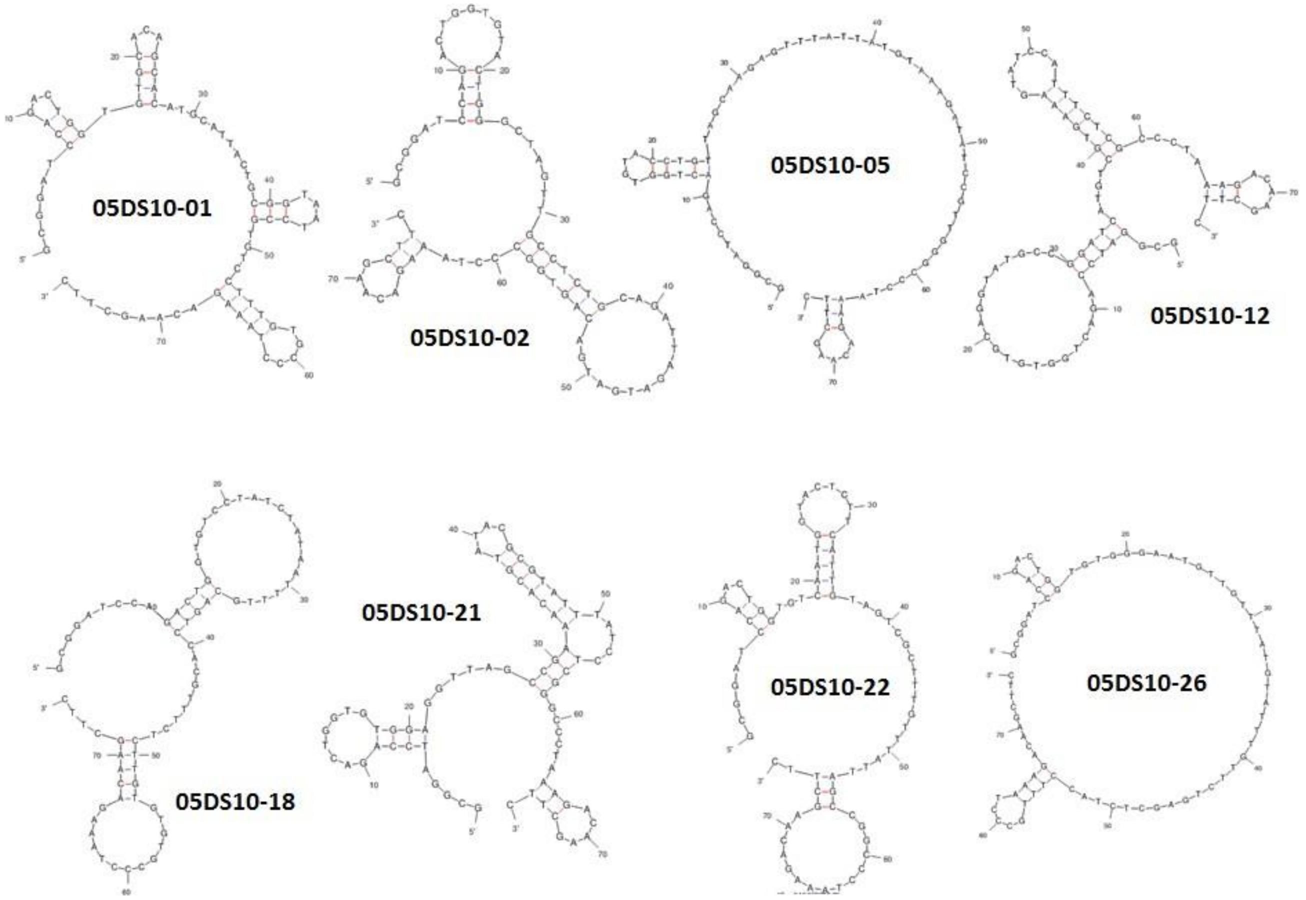

Supplementary Figure S5. Minimum free energy (MFE) structure drawings (predicted by mfold software using the ionic conditions of the SB and a folding temperature of 37°C) of the eight high-affinity ssDNA aptamers specific to PCBP-2 whose functional analysis is shown in Figure 5. The free energies of the MFE depicted are: 05DS10-01, -5.31 kcal/mol; 05DS10-02, -4.74 kcal/mol; 05DS10-05, -1.35 kcal/mol; 05DS10-12, -3.18 kcal/mol; 05DS10-18, -2.84 kcal/mol; 05DS10-21, -2.46 kcal/mol; 05DS10-22, -2.13 kcal/mol; 05DS10-26, -1.75 kcal/mol.

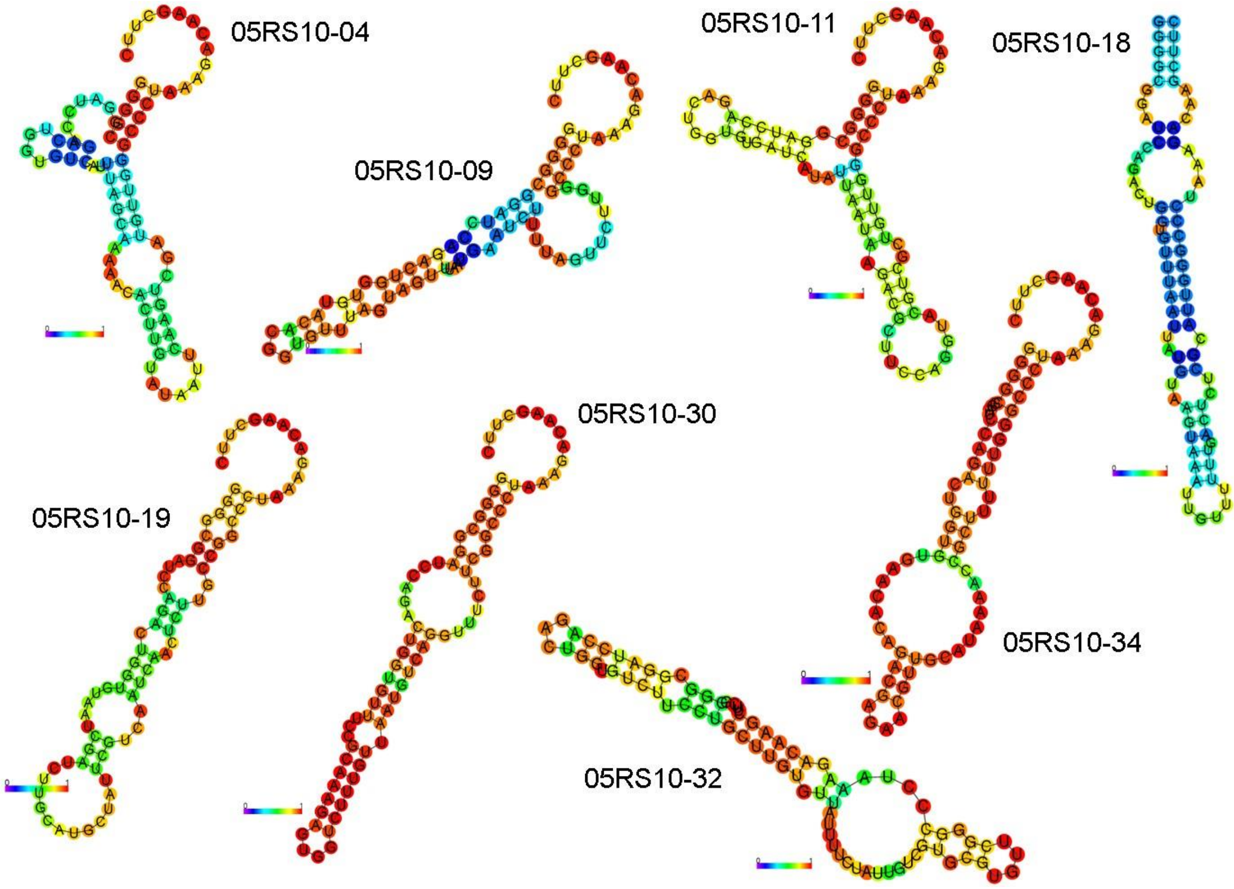

Supplementary Figure S6. Minimum free energy (MFE) structure drawings (predicted by RNAfold software) encoding base-pair probabilities of the eight high-affinity RNA aptamers specific to PCBP-2 whose functional analysis is shown in Figure 6. The free energies of the MFE depicted are: 05RS10-04, -15.10 kcal/mol; 05RS10-09, -14.10 kcal/mol; 05RS10-11, -16.50 kcal/mol; 05RS10-18, -10.80 kcal/mol; 05RS10-19, -15.10 kcal/mol; 05RS10-30, -23.40 kcal/mol; 05RS10-32 -16.60 kcal/mol; 05RS10-34, -17.40 kcal/mol.

A

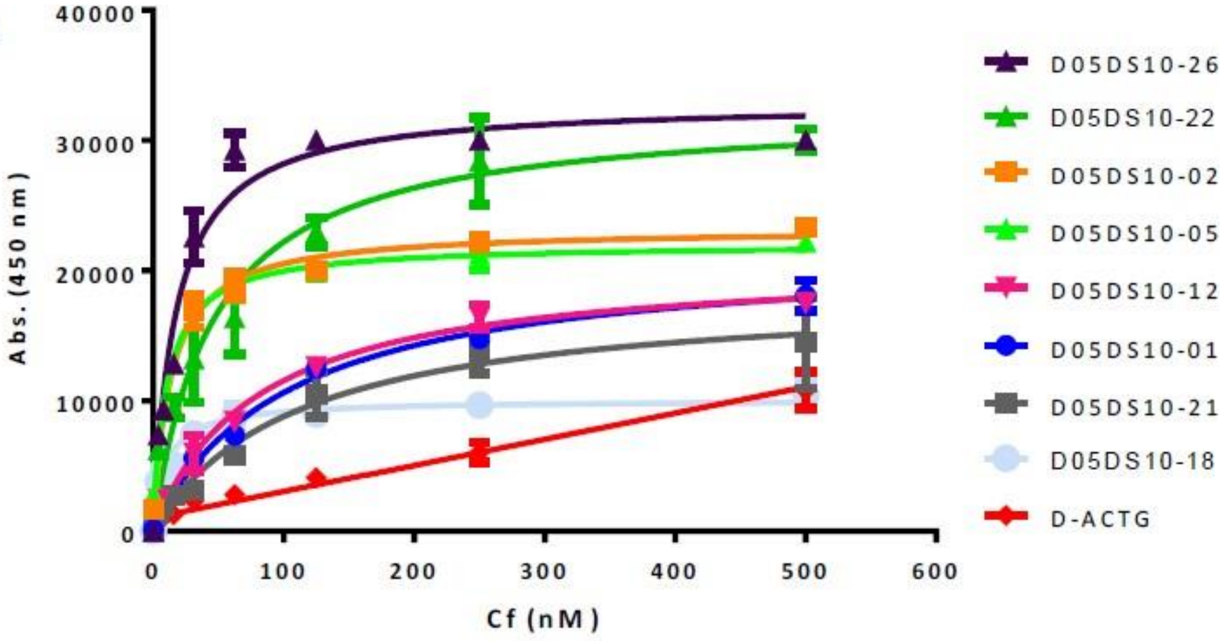

B

	Kd (nM)	SD	Bmax (Abs. 450 nm)	SD	R^2
D05DS10-26	**16.9**	1.8	**3.3x10^{04}**	8.2x10^{02}	**0.97**
D05DS10-22	**45.9**	7.2	**3.2x10^{04}**	1.5x10^{03}	**0.95**
D05DS10-02	**13.2**	2.1	**2.3x10^{04}**	5.2x10^{02}	**0.98**
D05DS10-05	**10.5**	2.5	**2.2x10^{04}**	6.3x10^{02}	**0.96**
D05DS10-01	**102.5**	10.1	**2.2x10^{04}**	7.5x10^{02}	**0.99**
D05DS10-12	**79.4**	9.9	**2.1x10^{04}**	8.4x10^{02}	**0.97**
D05DS10-21	**109.5**	21.7	**1.8x10^{04}**	1.4x10^{03}	**0.93**
D05DS10-18	**9.9**	1.2	**1.0x10^{04}**	2.6x10^{02}	**0.95**

	Slope	R^2
D-ACTG (Neg. Control)	20.0	0.98

Supplementary Figure S7. Characterization of high affinity ssDNA individual aptamers present in the last SELEX round, by means of colorimetric ELONA. The affinity curves obtained for the eight selected individual aptamers (**A**) showed that the best fit curve corresponded to a *One site - specific binding* model (with R^2 values in the range 0.93-0.99) in all cases, from which the *Kd* and *Bmax* values were derived (**B**). In parallel, D-ACTG molecule used as a negative control (see text for details) could only be adjusted to a linear regression model, thus showing non-specific binding to PCBP-2.

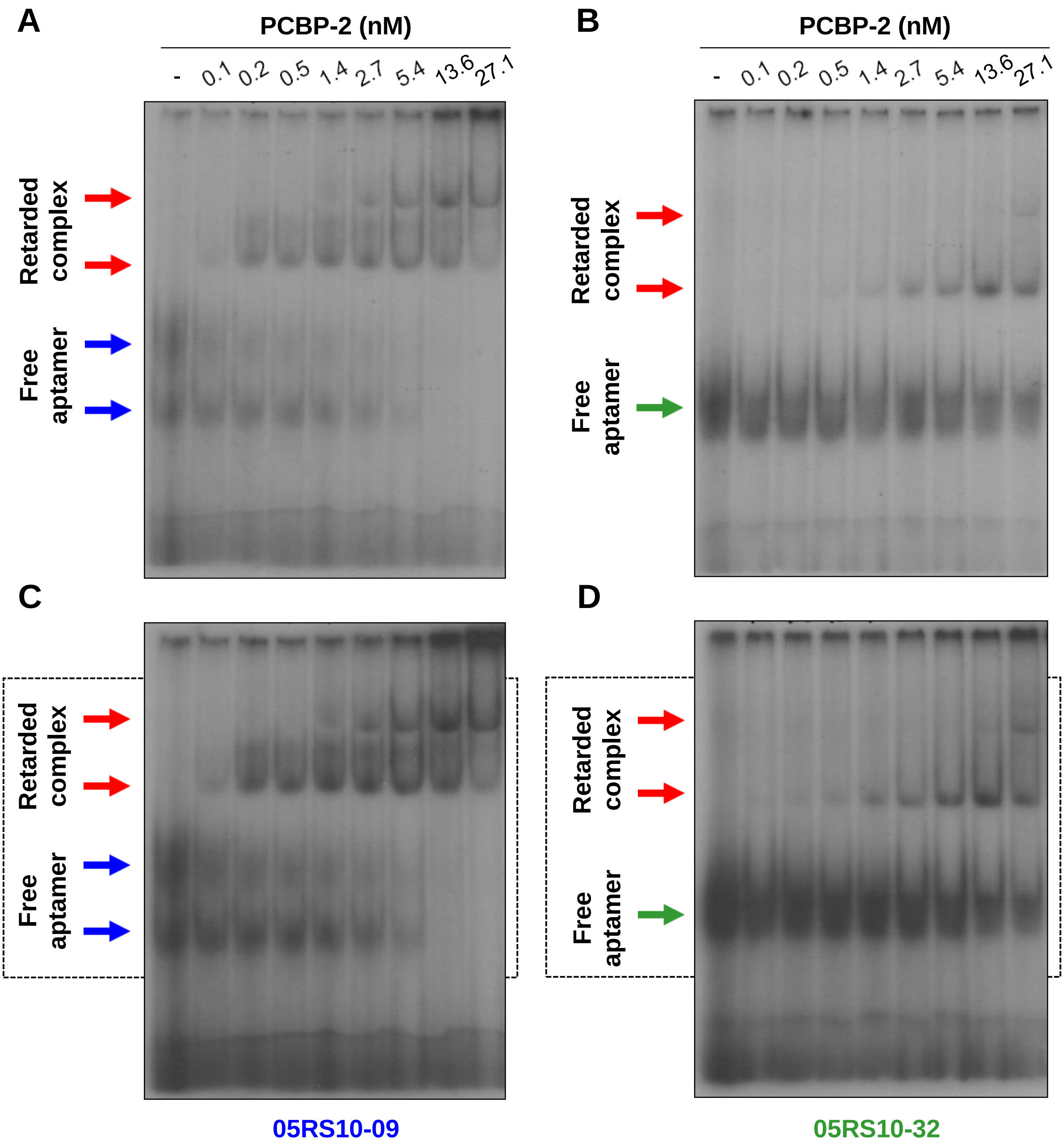

Supplementary Figure S8. Full-length EMSA gels showing the aptamer–PCBP-2 complexes formed by two high affinity RNA aptamers: 05RS10-09 (**A, C**) and 05RS10-32 (**B, D**). Short (16 h: **A**, **B**) and long (80 h: **C**, **D**) exposure times at -80˚C were used in both cases. Boxes in panels **C** and **D** correspond to panels **A** and **B** of Figure 7.
